# Phosphorylation of protein kinase Cδ Tyr311 positively regulates thromboxane generation in platelets

**DOI:** 10.1016/j.jbc.2021.100720

**Published:** 2021-04-28

**Authors:** John C. Kostyak, Benjamin Mauri, Akruti Patel, Carol Dangelmaier, Haritha Reddy, Satya P. Kunapuli

**Affiliations:** 1 Sol Sherry Thrombosis Research Center, Temple University School Lewis M Katz School of Medicine, Philadelphia, Pennsylvania, USA; 2 Department of Physiology, Temple University School Lewis M Katz School of Medicine, Philadelphia, Pennsylvania, USA; 3 Department of Pharmacology, Temple University School Lewis M Katz School of Medicine, Philadelphia, Pennsylvania, USA

**Keywords:** platelet, protein kinase C, thrombosis, hemostasis, tyrosine, CRP, collagen-related peptide, ERK1/2, extracellular signal-regulated protein kinase, GPVI, glycoprotein VI, KI, knock-in, PAR, protease-activated receptor, PKC, protein kinase C

## Abstract

Platelets are key mediators of physiological hemostasis and pathological thrombosis, whose function must be carefully balanced by signaling downstream of receptors such as protease-activated receptor (PAR)4. Protein kinase C (PKC) is known to regulate various aspects of platelet function. For instance, PKCδ is known to regulate dense granule secretion, which is important for platelet activation. However, the mechanism by which PKCδ regulates this process as well as other facets of platelet activity is unknown. We speculated that the way PKCδ regulates platelet function may be because of the phosphorylation of tyrosine residues on PKCδ. We investigated phosphorylation of PKCδ following glycoprotein VI-mediated and PAR4-mediated platelet activation and found that Y311 is selectively phosphorylated when PAR4 is activated in human platelets. Therefore, we generated PKCδ Y311F knock-in mice, which are viable and have no gross abnormalities. However, PKCδY311F mice have significantly enhanced tail-bleeding times compared with WT littermate controls, which means hemostasis is interrupted. Furthermore, PKCδY311F mice exhibit longer time to carotid artery occlusion compared with WT control using a ferric chloride *in vivo* thrombosis model, indicating that the phosphorylation of PKCδ Y311 is prothrombotic. Washed platelets from PKCδY311F mice have reduced reactivity after stimulation with a PAR-4 agonist indicating its importance in platelet signaling. The phenotype observed in Y311F mouse platelets is because of reduced thromboxane generation, as an inhibitor of thromboxane generation equalizes the PKCδY311F platelet response to that of WT. Therefore, phosphorylation of PKCδ on Y311 is important for regulation of platelet function and specifically thromboxane generation, which reinforces platelet activation.

Platelets are anucleate cells that travel close to the vascular wall and respond to vascular damage (1). They are key mediators of both physiological hemostasis and pathological thrombosis (2, 3). Platelet signaling and activity exists in a delicate balance between hemorrhage and occlusion. Weak platelet reactivity can lead to bleeding, whereas strong platelet reactivity can lead to thrombosis. It is, therefore, important to understand the nuances of signaling that regulate platelet activation.

Primary platelet signaling, such as that which is initiated by glycoprotein VI (GPVI) engagement of collagen, leads to granule release and production of thromboxane (4). Thromboxane and the granular contents feedback on the platelet in an autocrine and paracrine manner to reinforce the original signal. These secondary signals also serve to recruit new platelets to the site of injury. Pharmacological manipulation of the secondary signals induced by adenosine diphosphate, which is released from dense granules and binds the surface receptors P2Y1 and P2Y12, and thromboxane are targets of antiplatelet therapy (5).

Platelets express many types of receptors on their surface including GPVI and protease-activated receptors (PAR) (6). PARs expressed on the platelet surface are activated by proteolytic cleavage by thrombin (7, 8). *In vitro*, PARs can be activated by peptides that correspond to their specific tethered ligands such as AYPGKF for PAR4 and SFLLRN for PAR1. Human platelets express PAR1 and PAR4, whereas mouse platelets express PAR2, PAR3, and PAR4 (9, 10, 11). PAR-induced platelet activation is caused by a host of platelet signaling pathways that lead to shape change, granular secretion, and aggregate formation. One protein involved in this pathway is protein kinase C (PKC) (12).

PKC is a member of the serine/threonine family of protein kinases, and the isoforms of PKC are categorized according to their cofactor requirements. Conventional PKC isoforms include α, βI, βII, and γ and require calcium and diacylglycerol for their activation. Novel PKC isoforms include δ, ε, θ, and η and require only diacylglycerol for activation. Atypical PKCs require phospholipids for their activation and include the isoforms ι, λ, and ζ (13). We, and others, have described the role of several PKC isoforms in platelet activation including PKCδ (12, 14, 15, 16, 17, 18, 19).

PKCδ is a novel PKC that is involved in the progression of several disease states including cancer, diabetes, heart disease, and sepsis (20, 21, 22, 23, 24). Furthermore, PKCδ is an important mediator of platelet production and activation. We previously demonstrated that PKCδ potentiates PAR4-mediated platelet reactivity, while it abrogates GPVI-mediated platelet reactivity in both human and mouse platelets (12, 16, 25). PKCδ is phosphorylated on a number of tyrosine residues which serve to regulate the kinase. Mouse PKCδ contains 19 tyrosine residues including Y311, which is located in the hinge region and regulates PKCδ conformation and is important for kinase activity and subcellular localization (26, 27). However, the impact of Y311 phosphorylation on PKCδ function in platelets has not been investigated.

In this report, we show that Y311 is differentially phosphorylated by GPVI or PAR-4 agonists. We produced a knock-in mouse with a mutated PKCδ Y311 that cannot be phosphorylated (Y311F). We provide evidence that suggests PKCδ Y311 is important for thromboxane generation in mouse platelets, which impacts *in vivo* regulation of hemostasis and thrombosis.

## Results

### Phosphorylation of tyrosine 311 on PKCδ

**Figure 1:**
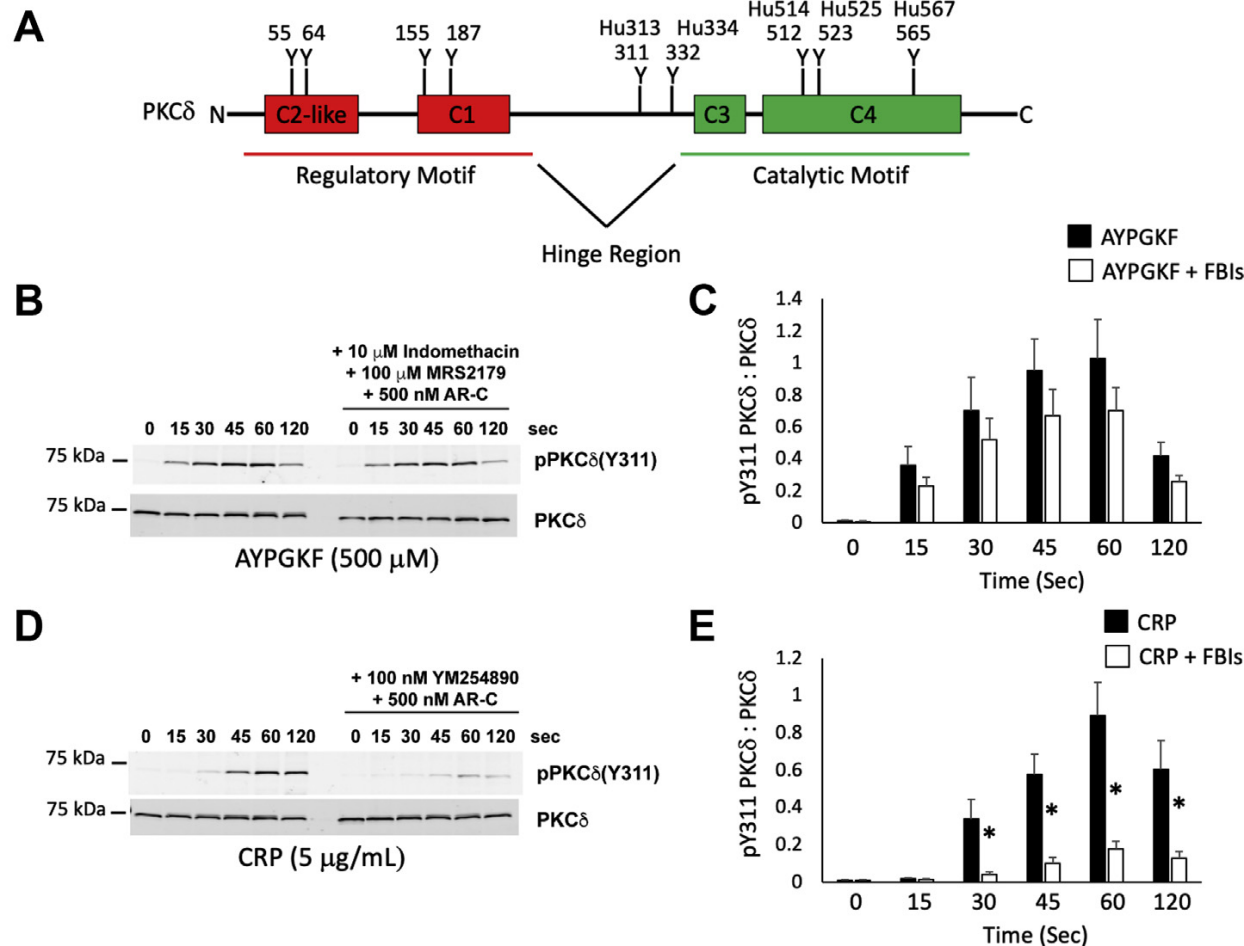
**Phosphorylation of Y311 on PKCδ is agonist dependent.***A*, schematic showing the locations of several tyrosine phosphorylation sites on PKCδ. Mouse and human sites are listed. An “Hu” prior to the number denotes human. *B*, western blots showing Y311 phosphorylation of PKCδ following stimulation of platelets with 500 μM AYPGKF with or without 10 μM Indomethacin, 100 μM MRS2179 and 500 nM AR-C69931MX. The combination of indomethacin, MRS2179 and AR-C69931MX are referred to as feedback inhibitors (FBIs). Washed human platelets were stimulated with 500 mM AYPGKF for the indicated time and the resulting platelet proteins were separated *via* SDS-PAGE. *C*, quantification of fluorescent band intensity from “*B*” expressed a ratio of pY311 to total PKCδ. *D*, western blots showing Y311 phosphorylation of PKCδ following stimulation (3 min) of human platelets with 5 μg/ml CRP with or without 100 nM YM-254890 and 500 nM AR-C69931MX. YM-254890 and AR-C69931MX are FBIs in this experiment. *E*, quantification of “*D*” expressed as a ratio of pY311 to total PKCδ, which was used to assess loading. ∗*p* < 0.05, n = 5. CRP, collagen-related peptide; PKC, protein kinase C.

We have previously shown that PKCδ differentially regulates platelet reactivity (16). A potential explanation for this could be that phosphorylation of specific tyrosine residues on PKCδ could determine its action. As one can see in Figure 1*A*, PKCδ has a number of tyrosine residues that reside within its different domains. One such residue is Y311 located within the hinge region. An analysis of Y311 phosphorylation using an agonist for PAR4 (AYPGKF) and an agonist for GPVI (collagen-related peptide [CRP]) showed that Y311 maybe an important residue for PKCδ function. We observed that phosphorylation of Y311 is rapid and pronounced in human platelets in response to AYPGKF (Fig. 1, *B* and *C*). Whereas the response to CRP is slower though just as pronounced 60 s after agonist addition (Fig. 1, *D* and *E*). Interestingly, when feedback is limited using indomethacin to block thromboxane production, MRS2179 to antagonize P2Y1, and AR-C69931MX to antagonize P2Y12, we observed very little change in Y311 phosphorylation in response to AYPGKF indicating that Y311 is phosphorylated downstream of the PAR4 receptor. Conversely, Y311 phosphorylation was greatly reduced in response to CRP when feedback was inhibited using AR-C69931MX and YM254890, which is a G_αq_ inhibitor. This suggests that the Y311 phosphorylation of PKCδ we observe in response to CRP is almost entirely because of feedback. Specifically, YM254890 would block the feedback response through P2Y1 and the thromboxane prostanoid receptor, which are both coupled to G_αq_. Therefore, Y311 may be a crucial phosphorylation site that dictates PKCδ function and may help explain the differential regulation of platelet response that we previously reported in PKCδ null mice. To expand our studies of Y311 on PKCδ, we produced a PKCδ Y311F mouse line.

### Production of PKCδ Y311F mice

**Figure 2:**
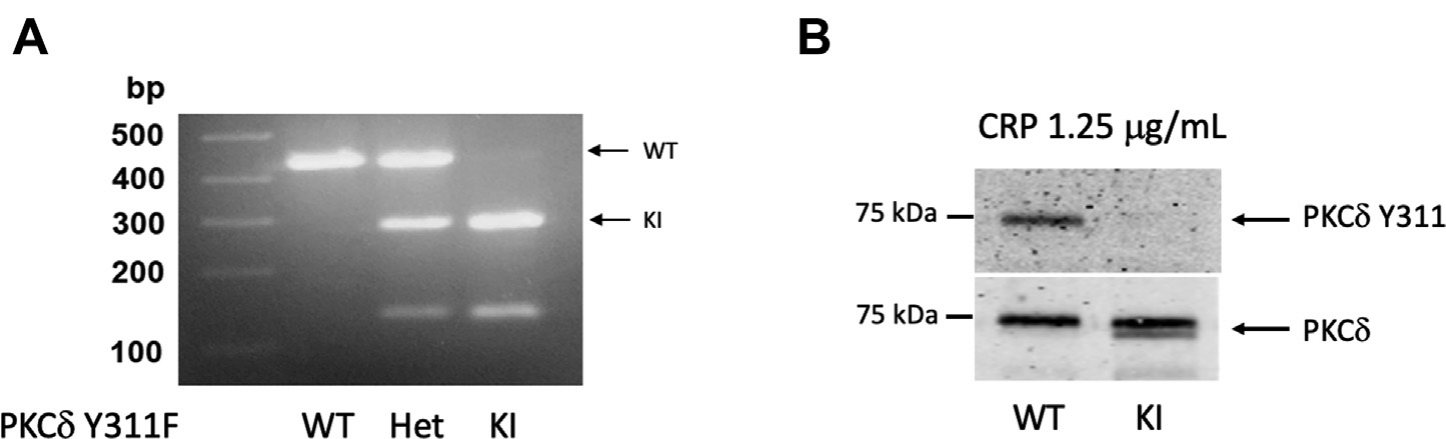
**Production of PKCδ Y311F mice.***A*, agarose gel of PCR products following SSpI restriction enzyme digest to identify PKCδ Y311F mice. WT PCR products remain uncleaved and are 432 bp, while heterozygotes (het) have the full length 432 bp product and the cleaved 147 bp and 285 bp products. The homozygous samples contain only the cleaved 147 bp and 285 bp products. *B*, western blot following immunoprecipitation of PKCδ from Y311F and WT control platelets. Washed platelets were stimulated with 1.25 μg/ml CRP for 3 min. The resulting platelet proteins were subjected to immunoprecipitation targeting PKCδ. The immunoprecipitated protein was resolved *via* SDS-PAGE, and the membranes were probed using an antibody against Y311 of PKCδ. Total PKCδ was used to assess loading. CRP, collagen-related peptide; PKC, protein kinase C.

We previously reported that Y311 on PKCδ is phosphorylated following stimulation of PAR4 in mouse platelets and that this phosphorylation is dependent on the Src family kinase, Lyn (28, 29). To better understand the functional role of phosphorylation of Y311 on PKCδ, we produced PKCδ Y311F knock-in (KI) mice that cannot be phosphorylated on Y311, using the CRISPR/Cas9 technique. Genotyping was conducted using PCR with the following primers: Prkcd I9F 5’-GGTTCTGCCCTCTGACGTCATCG-3’ and Prkcd I10R 5’-GCTAGGCAAGGGGCCTTGGAG. The resultant PKCδ Y311F knock-in product has a sequence that can be cleaved by the restriction enzyme SspI, whereas the WT product cannot be cleaved. Therefore, the WT mice will have the full-length product, which is 432 bp, the heterozygous mice will have the full-length product of 432 bp and the cleaved products of 147 bp and 285 bp, and the KI will have only the cleaved products of 147 bp and 285 bp (Fig. 2*A*). We confirmed the loss of Y311 phosphorylation of PKCδ using washed mouse platelets stimulated with CRP, which is an agonist for GPVI (Fig. 2*B*). As one can see the WT platelets displayed strong phosphorylation of PKCδ Y311, whereas we were unable to detect any phosphorylation of Y311 in the platelets isolated from KI mice. PKCδ Y311F mice breed normally and produce pups at expected Mendellian ratios. Furthermore, the mice display no gross abnormalities, gain weight normally, and have normal blood cell counts (Table 1).

**Table 1:** Blood cell counts in PKCδY311F and WT littermate control mice

Parameter	WT	PKCδ Y311F
WBC (10^6^/ml)	10.22 ± 1.40	9.78 ± 2.15
NE (10^6^/ml)	1.10 ± 0.32	1.06 ± 0.16
LY (10^6^/ml)	8.58 ± 1.31	8.31 ± 1.92
Plt (10^6^/ml)	721 ± 37.58	715 ± 78.69
MPV (fl)	4.15 ± 0.10	4.05 ± 0.06

LY, lymphocyte; MPV, mean platelet volume; NE, neutrophil; Plt, platelet; WBC, white blood cell.

### Hemostasis and thrombosis are perturbed in PKCδ Y311F mice

**Figure 3:**
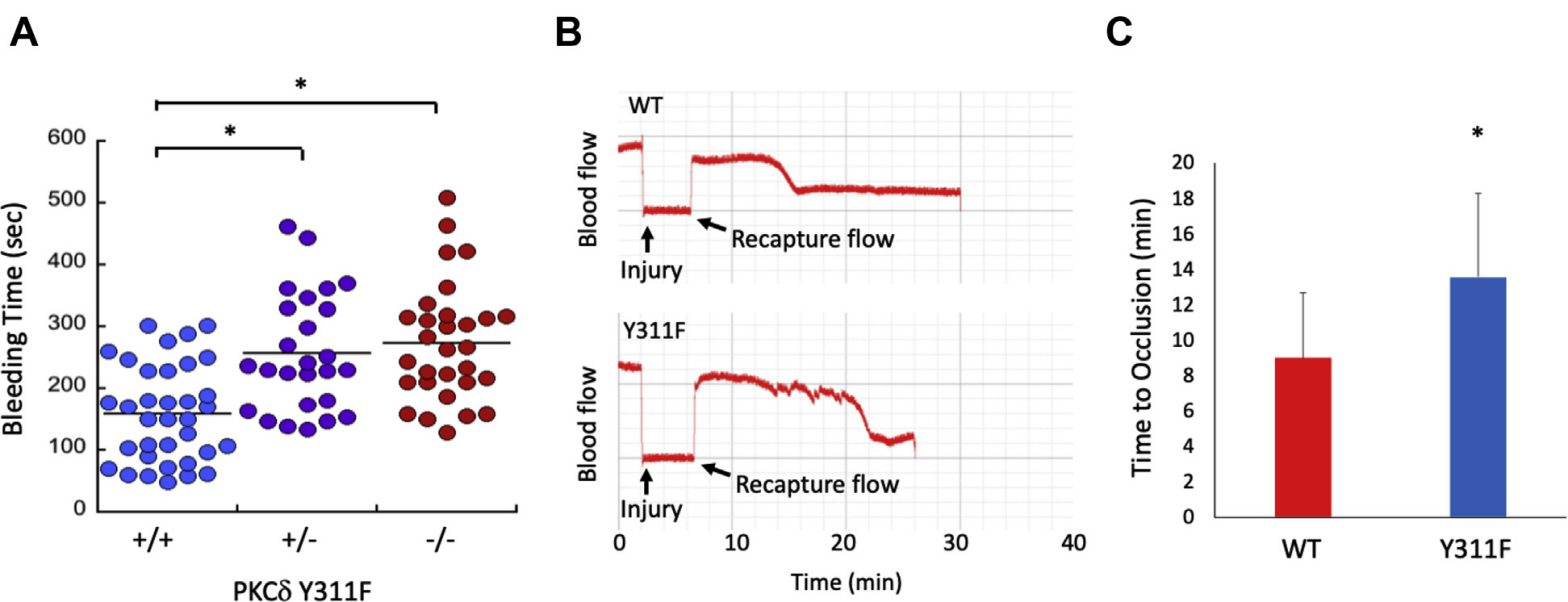
**Hemostasis and thrombosis are altered in PKCδ Y311F mice.***A*, tail bleeding times from WT (+/+), heterozygous (+/−), and PKCδY311F KI (−/−) mice. The distal 3 mm of the tail was clipped from mice aged 4 to 6 weeks and allowed to bleed into warm saline. The time until bleeding ceased was recorded. ∗*p* < 0.05. *B*, representative blood flow tracings from WT control and PKCδY311F Ki mice subjected to FeCl_3_ injury of the carotid artery. *C*, time to occlusion following vessel injury was recorded. ∗*p* < 0.05, n = 7. PKC, protein kinase C.

To determine the consequence of the PKCδ Y311F mutation on hemostasis *in vivo*, we performed tail bleeding experiments on WT, heterozygous, and homozygous PKCδ Y311F mice. Interestingly, both the heterozygous and the homozygous knock-in mice bled for significantly longer than the WT littermate control mice (Fig. 3*A*). This suggests that the Y311F point mutation disrupts hemostasis. To determine whether or not thrombosis is also altered, we injured the carotid artery of WT and PKCδ Y311F mice using the FeCl_3_ injury model. We found that time to vessel occlusion was significantly increased in the PKCδ Y311F KI mice compared with WT littermate control mice (Fig. 3, *B* and *C*). These data are consistent with our tail-bleeding data and taken together suggest that thrombosis and hemostasis are both inhibited by the mutation of Y311 on PKCδ.

### PAR4-mediated secretion is reduced in PKCδ Y311F mouse platelets

**Figure 4:**
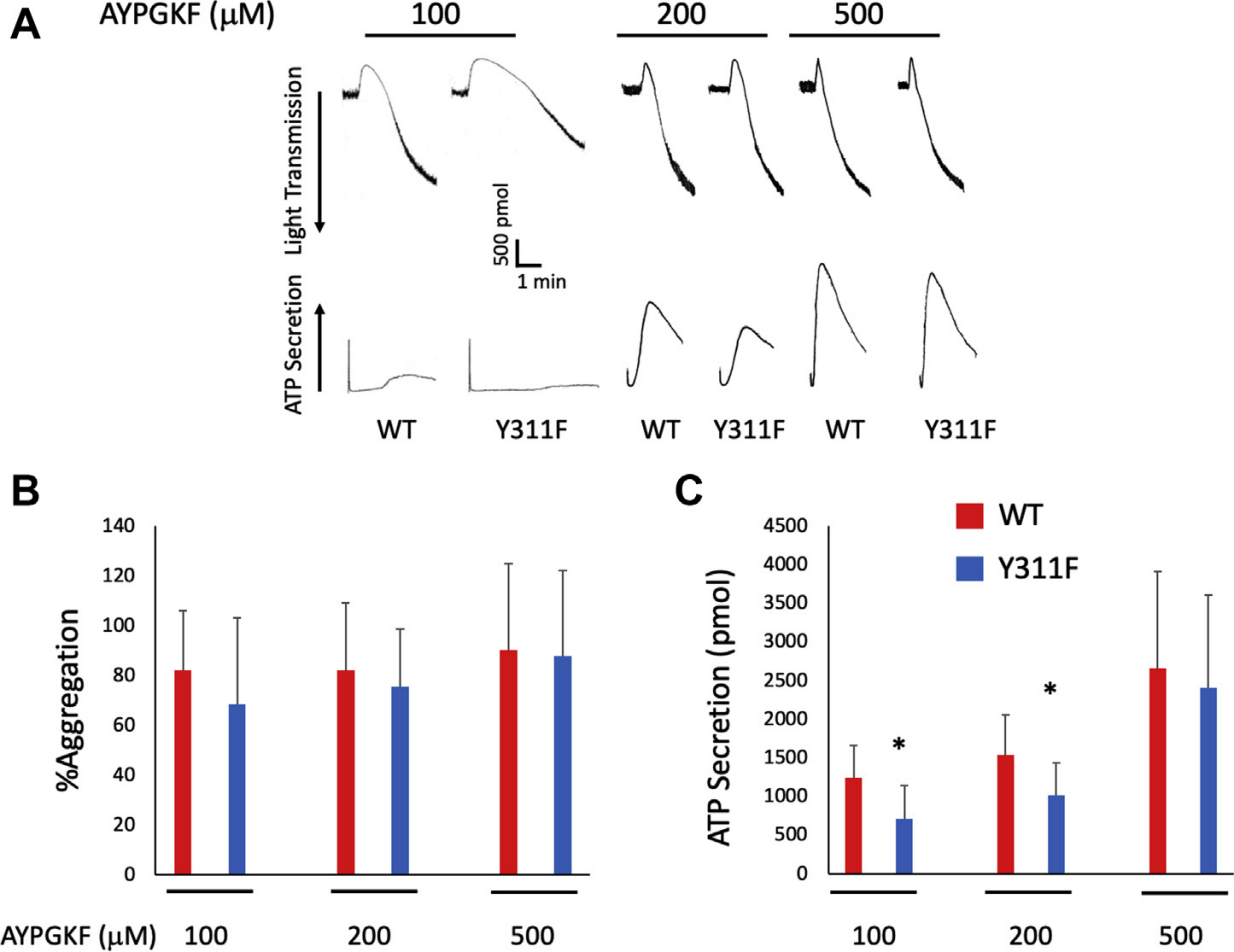
**PAR-4 induced activation is impaired in platelets from PKCδ Y311F mice.***A*, washed platelets from WT control and PKCδ Y311F mice were stimulated with the indicated concentration of AYPGKF, and aggregation and secretion were recorded. *B*, representative tracings that show aggregation was unaltered in PKCδ Y311F platelets stimulated with AYPGKF. *C*, ATP secretion from PKCδ Y311F platelets was diminished when stimulated with 100 μM and 200 μM AYPGKF compared with WT control platelets. ∗*p* < 0.05, n = 5. PAR, protease-activated receptor; PKC, protein kinase C.

Our data presented above suggests that Y311 on PKCδ is phosphorylated directly downstream of the PAR4 receptor. To determine how PKCδ Y311 phosphorylation impacts platelet reactivity and, therefore, hemostasis and thrombosis, we used washed platelets from Y311F mice and WT littermate controls and stimulated them with AYPGKF (a PAR-4 agonist). We monitored aggregation and ATP secretion following stimulation with three different concentrations of AYPGKF and found that ATP secretion following stimulation with either 100 μM or 200 μM AYPGKF is reduced in Y311F platelets compared with WT platelets (Fig. 4, *A*–*C*), although aggregation was unaltered. These data are in agreement with our *in vivo* data that suggest Y311F positively regulates platelet function.

### Y311 on PKCδ regulates thromboxane production

**Figure 5:**
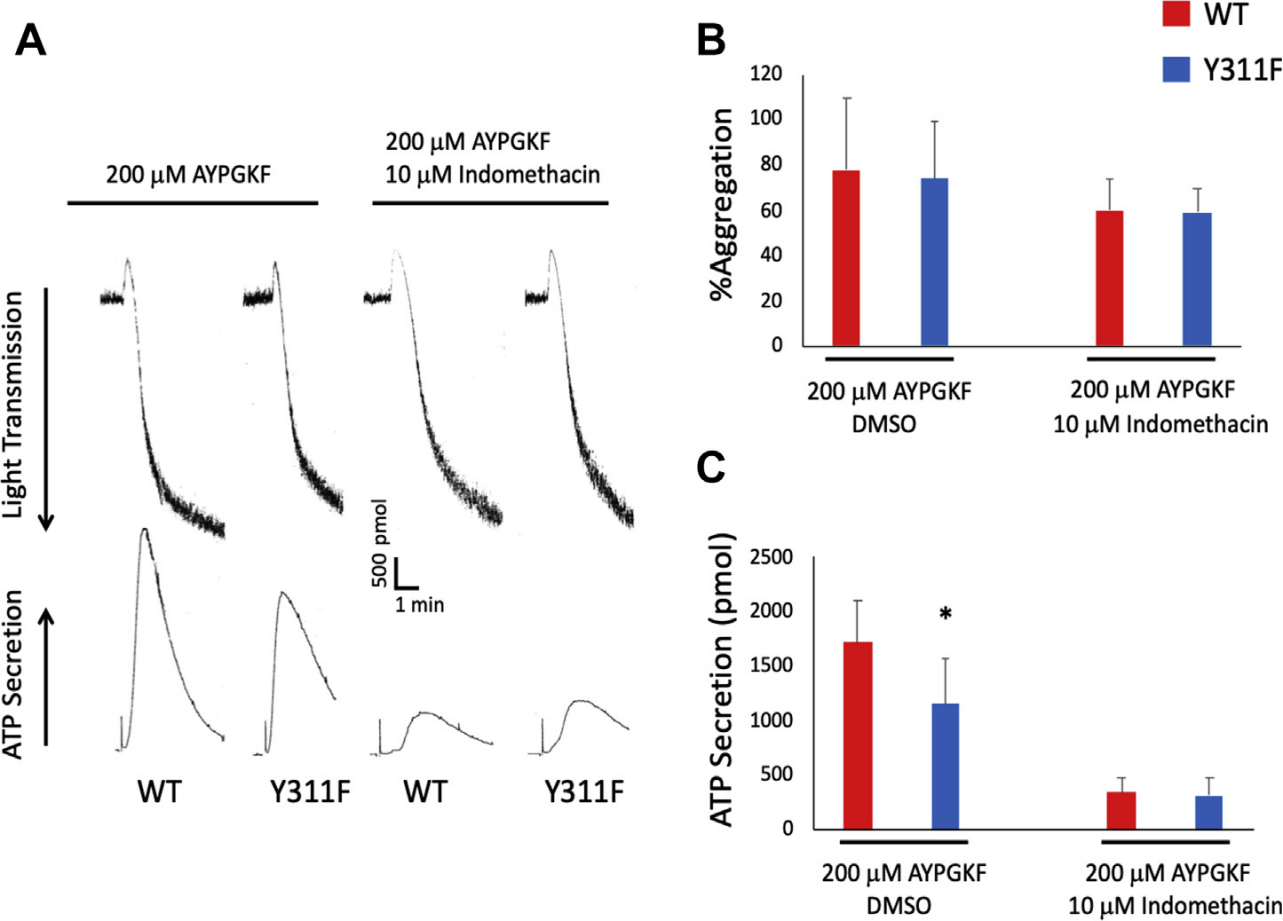
**Inhibition of thromboxane generation equalizes ATP secretion between Y311F and WT platelets.***A*, washed platelet from PKCδ Y311F or WT control mice were either pretreated with indomethacin to inhibit thromboxane production or dimethyl sulfoxide as a vehicle control. Platelets were then stimulated with 200 μg/ml AYPGKF, and aggregation and secretion were observed. Representative tracings are shown. *B*, aggregation was unaltered with indomethacin pretreatment. *C*, ATP secretion was reduced in Y311F platelets when vehicle control was added but was normalized in PKCδ Y311F platelets following indomethacin pretreatment. ∗*p* < 0.05 compared with WT, n = 5. PKC, protein kinase C.

Because we observe reduced platelet reactivity to low concentrations of AYPGKF, which is often driven by thromboxane production, we chose to investigate thromboxane generation in Y311F platelets. Thromboxane production occurs swiftly following platelet stimulation and drives platelet activation. Therefore, we pretreated washed platelets from Y311F mice and WT control mice with vehicle control (dimethyl sulfoxide) or indomethacin, an inhibitor of COX-2, which is part of the thromboxane production pathway, and stimulated them with AYPGKF. In agreement with our data presented above, dimethyl sulfoxide pretreatment resulted in reduced secretion of ATP in Y311F platelets compared with WT platelets. Interestingly, pretreatment with indomethacin results in no difference in ATP secretion between Y311F platelets and WT control platelets (Fig. 5, *A*–*C*). These data strongly suggest that thromboxane production is responsible for the phenotype observed in Y311F mice and that Y311 phosphorylation is integral for thromboxane production in platelets.

**Figure 6:**
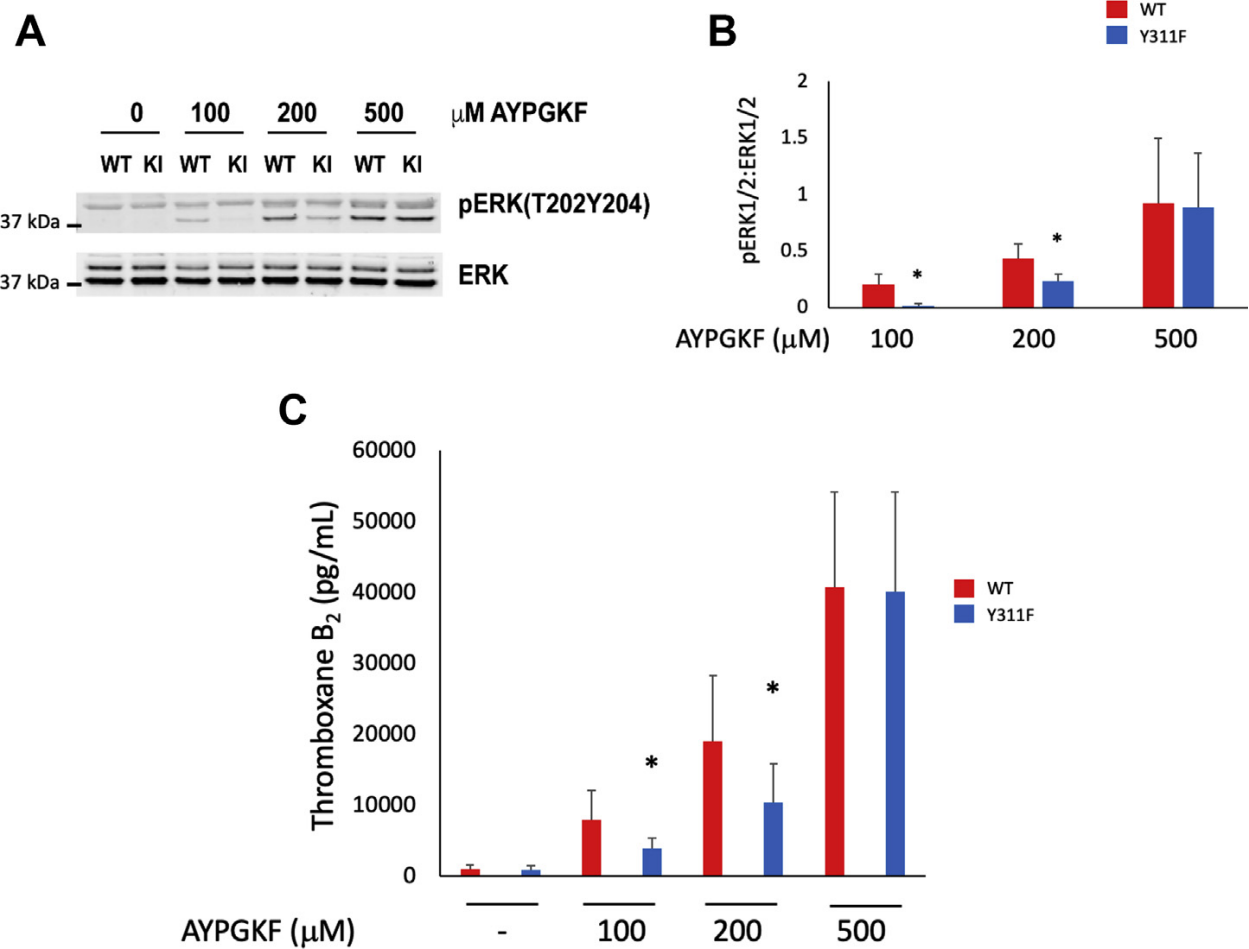
**Y311 on PKCδ regulates thromboxane generation.***A*, representative western blots of platelet proteins from WT and Y311F mice. Platelets were stimulated with the indicated concentration of AYPGKF, and the membranes were probed for phosphorylated ERK1/2 and total ERK1/2, which was used to assess loading. *B*, quantification from multiple experiments described in *B*, expressed as a ratio of phosphorylated ERK1/2 to total ERK1/2. *C*, washed platelets from WT control and PKCδ Y311F mice were stimulated with the indicated concentration of AYPGKF for 3 min under stirring conditions. Thromboxane generation was assessed using a kit from Enzo Scientific. ∗*p* < 0.05, n = 5. ERK1/2, extracellular signal-regulated protein kinase; PKC, protein kinase C.

We have previously demonstrated that extracellular signal-regulated protein kinase (ERK1/2) phosphorylation regulates thromboxane generation (30, 31, 32). Therefore, we evaluated ERK1/2 phosphorylation in WT and Y311F mouse platelets stimulated with AYPGKF for 3 min *via* SDS-PAGE. We found that ERK1/2 phosphorylation was reduced in Y311F platelets stimulated with low concentrations of AYPGKF (Fig. 6, *A* and *B*). These data suggest that Y311 phosphorylation on PKCδ may regulate thromboxane generation. To investigate this potential mechanism, we stimulated washed platelets from Y311F mice and platelets from WT control mice with AYPGKF. We snap froze the samples at the conclusion of the experiment and used a kit from Enzo scientific to measure thromboxane production. We determined that thromboxane production was indeed reduced in samples collected using platelets from Y311F mice compared with those collected from WT platelets at lower concentrations of AYPGKF (Fig. 6*C*). These results, coupled with our data presented in Figure 5, strongly suggest that Y311 on PKCδ is an important mediator of thromboxane generation.

## Discussion

**Figure 7:**
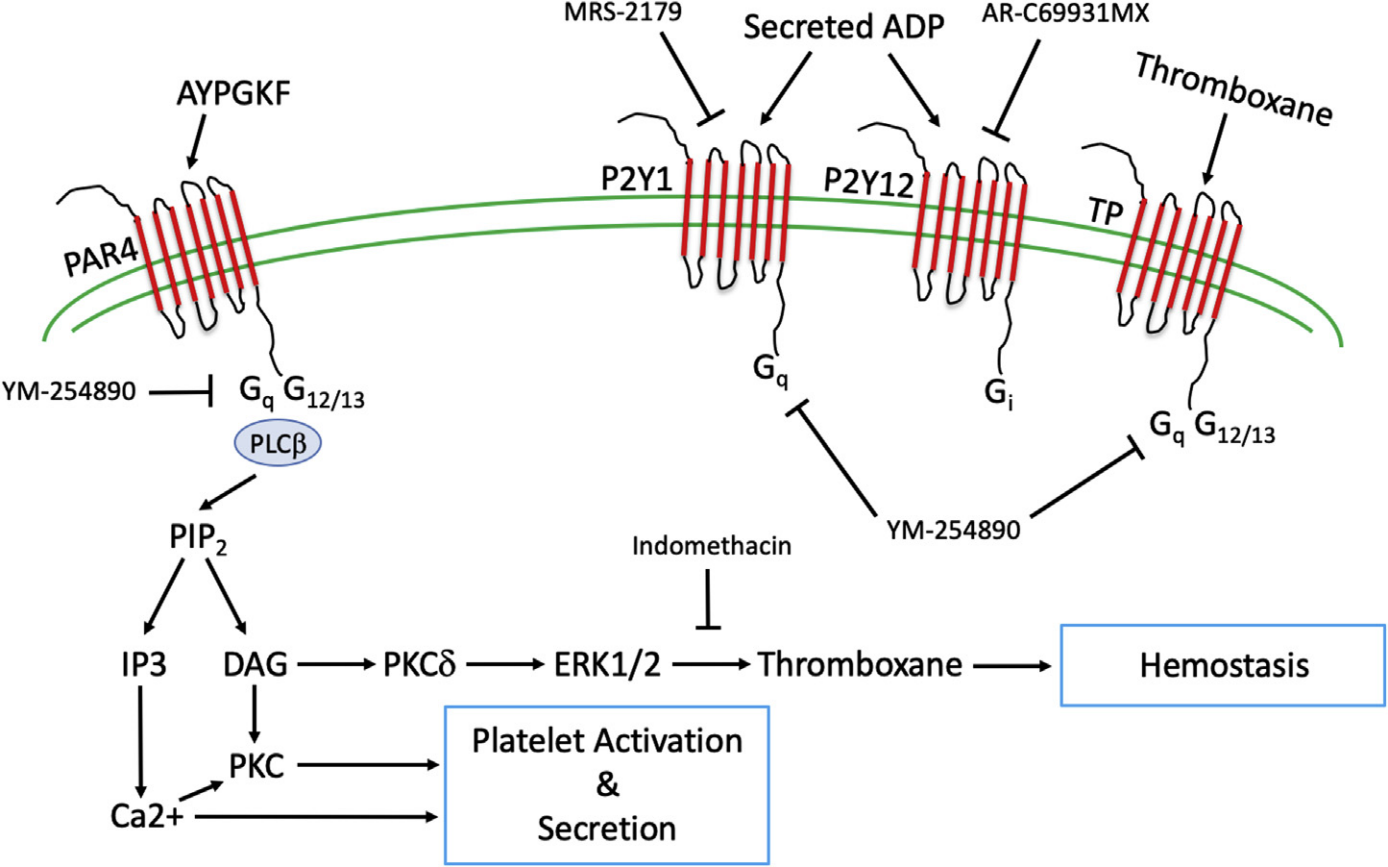
**Schematic representation of Y311 phosphorylated PKCδ signaling in the platelet.** Inhibitors and antagonists used during the collection of our data, as well as their targets, are also listed. Activation of PAR4 *via* AYPGKF leads to PLCβ activation and subsequent IP3 and DAG production. PKCδ responds to DAG and its activation results in ERK1/2 phosphorylation and thromboxane generation. Our data, presented in this manuscript, demonstrate that phosphorylation of Y311 on PKCδ regulates ERK1/2 phosphorylation and thromboxane generation. DAG, diacylglycerol; ERK1/2, extracellular signal-regulated protein kinase; PAR, protease-activated receptor; PKC, protein kinase C.

In this report, we demonstrate that Y311 phosphorylation on PKCδ is important for thromboxane production and is phosphorylated directly downstream of PAR4, but not directly downstream of GPVI. We show that platelets from Y311F mice have reduced reactivity compared with platelets from WT mice when G Protein coupled receptors are stimulated. We demonstrate that this phenotype is also observed using *in vivo* models of hemostasis and thrombosis. The mechanism behind reduced platelet reactivity and impaired hemostasis and thrombosis is inhibited thromboxane production as we can reverse the phenotype observed in Y311F platelets *via* preincubation with indomethacin, an inhibitor of thromboxane production. A summary of this mechanism is presented in Figure 7 along with the inhibitors and antagonists we used to isolate the signaling Y311 phosphorylated PKCδ is involved in.

Y311 phosphorylation on PKCδ regulates thromboxane generation. In this report, we demonstrate that PAR4-dependent platelet dense granule secretion is reduced and that thromboxane generation is reduced when either 100 μM or 200 μM AYPGKF is used as an agonist. Further, when using indomethacin, which inhibits thromboxane production, before stimulation with AYPGKF, the phenotype observed in PKCδY311F platelets and described above is normalized. These data strongly suggest that Y311 on PKCδ is involved in thromboxane generation.

Given our previous data that PKCδ differentially regulates platelet activation and that our Western blot data in Figure 1 shows differential phosphorylation of Y311 on PKCδ, we hypothesized that we would observe reduced reactivity to AYPGKF in platelets from Y311F mice, which our data supports (12, 16, 25). PKCδ is tyrosine phosphorylated on a number of different sites within conserved and variable regions which makes PKCδ unique among PKCs (27, 33, 34, 35). Tyrosine phosphorylation is known to influence PKCδ activation and conformation. Specifically, Y311 on PKCδ lies within the hinge region of the kinase, which is one of the variable regions (V4) that are poorly conserved across PKCs (36). *In vitro* assays have demonstrated that Y311 on PKCδ is a target for Src, which may enhance target specificity for PKCδ (37). Therefore, Src phosphorylation of Y311 on PKCδ may be responsible for directing PKCδ to a target that is important for thromboxane production downstream of PAR4 and likely any G Protein coupled receptor that is coupled to G_αq_ based on our data in Figure 1. That exact target is unknown at this time, but it would be worthwhile to elucidate.

PKCδ Y311 phosphorylation occurs downstream of G_αq_. In Figure 1, we demonstrate that Y311 on PKCδ is phosphorylated when either an agonist of PAR4 or GPVI is used. However, when feedback is blocked downstream of PAR4, Y311 phosphorylation is reduced only a minor and statistically insignificant amount. When feedback is blocked downstream of GPVI using an inhibitor of G_αq_, very little Y311 phosphorylation remains, which strongly suggests that Y311 phosphorylation is G_αq_ dependent. A similar experiment using the G_αq_ inhibitor YM254890 and stimulating platelets with a PAR4 agonist would be inconclusive as no platelet aggregation or secretion would occur because PAR4 is coupled to G_αq_.

PKCδ Y311 phosphorylation is important for *in vivo* platelet function. We observed that hemostasis was inhibited, as tail bleeding times were enhanced in PKCδY311F mice. This is in agreement with our data that demonstrated reduced thromboxane production in Y311F mouse platelets. This would be akin to a human taking aspirin, which inhibits COX-2 and subsequent thromboxane production and is associated with bleeding. We also demonstrated that thrombosis was inhibited, as the time to carotid artery occlusion was inhibited following injury in PKCδY311F mice compared with WT mice. This is in contrast to our previous report in which we show that thrombosis is unaltered in PKCδ knockout mice (12). The likely explanation for this discrepancy is that PKCδ has many tyrosine residues that can be phosphorylated, and they reside in various domains on PKCδ as outlined in Figure 1. Other tyrosine phosphorylation sites on PKCδ may influence its activation, conformation, or target specificity. In this report, we focused on one specific tyrosine residue on PKCδ. However, each individual tyrosine residue may regulate a different facet of PKCδ function. For instance, Y155 is located between the pseudo-substrate motif (autoinhibition) and the C1A domain (Fig. 1*A*), which serves as a diacylglycerol/phosphatidylserine/phorbol ester sensor and is responsible for a number of different PKCδ functions (27, 35, 38). We would hypothesize that phosphorylation of Y155 may direct PKCδ to a GPVI-specific substrate that is important for controlling GPVI signaling only and have no effect on PAR-4 mediated signaling, whereas Y311 phosphorylation is important for thromboxane production and occurs downstream of G_αq_. This could explain why we observe an increase in GPVI-mediated platelet activation in PKCδ knockout platelets, but reduced platelet activation in PKCδ Y311F platelets as reported here.

The data presented in this manuscript demonstrate that phosphorylation of specific tyrosines on PKCδ can greatly influence its function. Preventing phosphorylation of Y311 *via* genetic modification caused a decrease in thromboxane generation and subsequent platelet function both *in vitro* and *in vivo*. It will be interesting to see how other tyrosine sites regulate PKCδ function.

## Experimental procedures

### Antibodies and reagents

All reagents were purchased from Thermo Fischer Scientific unless otherwise stated. Collagen and Chronolume, used for the detection of secreted ATP, were purchased from Chrono-log Corporation. The CLEC-2 activating antibody was purchased from Biolegend. Odyssey blocking buffer and secondary antibodies IRDye 800CW goat anti-rabbit and IRDye 680LT goat anti-mouse were purchased from Li-Cor. CRP-XL was purchased from Dr Richard Farndale at the University of Cambridge. AYPGKF was purchased from GenScript. The restriction enzyme SspI was purchased from New England BioLabs.

### Animal housing and production

Mice were housed in a pathogen-free facility, and all animal procedures were approved by the Temple University Institutional Animal Care and Use Committee (protocol #4864). PKCδ Y311F mice were produced at the University of Connecticut Health Center on a fee for service basis.

### Preparation of human platelets

All studies involving human subjects were approved by the Temple University Institutional review board and abide by the Declaration of Helsinki principles. Human platelets were prepared as previously described and were resuspended to a final concentration of 2.0 × 108 cells/ml in N-2-hydroxyethylpiperazine-N’-2-ethanesulfonic acid-buffered (pH 7.4) Tyrode’s solution containing 0.2 U/ml apyrase (16).

### Preparation of mouse platelets

Mouse blood was collected, and platelets were isolated as previously described (39). The resulting platelets were counted using a Hemavet 950FS blood cell analyzer (Drew Scientific). Platelet counts were adjusted to a final concentration of 1.5 × 10^8^ cells/ml in N-2-hydroxyethylpiperazine-N’-2-ethanesulfonic acid-buffered (pH 7.4) Tyrode’s solution containing 0.2 U/ml apyrase.

### Platelet aggregation and ATP secretion

All platelet aggregation and secretion experiments were carried out using a lumi-aggregometer (Chrono-log) at 37 °C under stirring conditions. Platelet aggregation was measured using light transmission and ATP secretion was measured using Chrono-lume (a luciferin/luciferase reagent).

### Western blotting

Western blotting procedures were performed as described previously (39). Briefly, platelets were stimulated for the indicated time points in a Lumi-aggregometer with either a GPVI or a PAR-4 agonist. The reaction was stopped by precipitating the platelet proteins using 0.6 N HClO_4_ and washed with water before the addition of sample loading buffer. Platelet protein samples were then boiled for 5 min before resolution by SDS-PAGE and transfer to nitrocellulose membranes. The membranes were then blocked using Odyssey blocking buffer and incubated overnight with primary antibodies against the indicated protein. The membranes were then washed with Tris-buffered saline containing 0.1% Tween-20 before incubation with appropriate secondary antibodies for 1 h at room temperature. The membranes were washed again and imaged using a Li-Cor Odyssey infrared imaging system.

### Tail bleeding assay

Mouse tail bleeding was conducted as previously described (40). Mice aged 4 to 6 weeks were anesthetized before amputation of the distal 3 mm of the tail. The tail was then immersed in 37 °C saline, and bleeding was monitored. If bleeding continued for greater than 600 s, then bleeding was halted manually by applying pressure.

### Carotid artery injury

FeCl_3_ was used to injure the carotid artery as previously described (40). Mice aged 10 to 12 weeks were anesthetized, and the carotid artery was exposed. A baseline blood flow reading was obtained using a Transonic T402 flow meter. The carotid artery was injured using a 1 × 1 mm piece of filter paper saturated with 7.5% FeCl_3_ for 90 s. The filter paper was removed, and blood flow was recorded.

### Statistics

All statistical analysis was performed using Microsoft Excel, and data were analyzed using a Student’s *t* test, where *p* < 0.05 was considered statistically significant. All data are presented as means ± SD of at least three independent experiments.

## Data availability

All data are contained within this manuscript.

## Conflict of interest

The authors have no conflicts of interest to disclose.
